# FgUbiH Is Essential for Vegetative Development, Energy Metabolism, and Antioxidant Activity in *Fusarium graminearum*

**DOI:** 10.3390/microorganisms12102093

**Published:** 2024-10-20

**Authors:** Jinwen Ge, Huanchen Zhai, Lei Tang, Shuaibing Zhang, Yangyong Lv, Pingan Ma, Shan Wei, Yu Zhou, Xiaofu Wu, Yang Lei, Fengguang Zhao, Yuansen Hu

**Affiliations:** College of Biological Engineering, Henan University of Technology, Zhengzhou 450001, China; gejinwen2000@163.com (J.G.); tangleixs@163.com (L.T.); shbzhang@163.com (S.Z.); lvyangyong2011@163.com (Y.L.); mapingan@haut.edu.cn (P.M.); weishansd2014@163.com (S.W.); 18240509603@163.com (Y.Z.); 18779730547@163.com (X.W.); leiyang@haut.edu.cn (Y.L.); fgzhao@scut.edu.cn (F.Z.); hys308@126.com (Y.H.)

**Keywords:** *Fusarium graminearum*, deoxynivalenol, coenzyme Q, reactive oxygen species, mitochondria, hydroxylase, Fusarium head blight

## Abstract

Fusarium head blight in wheat is mainly caused by *Fusarium graminearum* and results in significant economic losses. Coenzyme Q (CoQ) is ubiquitously produced across organisms and functions as a hydrogen carrier in energy metabolism. While UbiH in *Escherichia coli* serves as a hydroxylase in CoQ biosynthesis, its role in phytopathogenic fungi is not well understood. This study explored the role of the hydroxylase FgUbiH in *F. graminearum*. Using a *FgUbiH* deletion mutant, we observed reduced hyphal growth, conidial production, germination, toxin synthesis, and pathogenicity compared to the wild-type. A transcriptome analysis indicated *FgUbiH*’s involvement in regulating carbohydrate and amino acid metabolism. Deletion of *FgUbiH* impaired mitochondrial function, reducing adenosine triphosphate synthesis and increasing reactive oxygen species. Additionally, genes related to terpene skeleton synthesis and aldehyde dehydrogenase were downregulated. Our results underscore the importance of FgUbiH in *F. graminearum*’s growth, toxin production, and energy metabolism, aiding in the development of strategies for disease management.

## 1. Introduction

The predominant contributor to Fusarium head blight (FHB) in wheat is *Fusarium graminearum*, which significantly reduces crop yield and produces diverse mycotoxins, causing significant economic losses and threatening food safety [1]. Currently, the primary management approach for FHB in agriculture involves chemical control, particularly pesticide application. However, a prolonged application of pesticides can induce resistance in pathogens, which necessitates the exploration of innovative fungicides, including the identification of new targets for fungicidal research by investigating their gene function.

Coenzyme Q (CoQ), also known as ubiquinone, serves as an antioxidant that prevents the oxidation of cellular proteins, lipids, and DNA [2]. As a free shuttle for electrons and hydrogen carriers within the membrane, it transfers active electrons from one complex to another, contributing to aerobic respiration [3]. CoQ is closely associated with energy generation and the fatty acid β-oxidation metabolic pathway in organisms [4,5]. The biosynthesis of CoQ is complex and differs between prokaryotes and eukaryotes. In *Escherichia coli*, CoQ8 biosynthesis involves a sequential process starting with decarboxylation and then C5-hydroxylation reactions, whereas, in *Saccharomyces cerevisiae*, CoQ6 biosynthesis starts with C5-hydroxylation and O-methylation, followed by decarboxylation [6]. CoQ biosynthesis requires one isopropylation, one decarboxylation, three hydroxylations, and three methylation reactions. The CoQ biosynthetic pathway has been comprehensively elucidated in *E. coli,* where the hydroxylation reactions are catalyzed by UbiH, UbiI, and UbiF [7,8,9]. These three proteins exhibit sequence identities ranging from 29 to 38% and are classified as class A flavoprotein monooxygenases [10]. In *S. cerevisiae*, the hydroxylation of CoQ biosynthesis involves CoQ6 and CoQ7. CoQ6 is classified as an A-type flavin monooxygenase and serves as a C-5 hydroxylase enzyme [11]. Conversely, CoQ7 functions as a ferrous monooxygenase and a C-6 hydroxylase [12]. However, the enzyme responsible for C-1 hydroxylation has yet to be identified.

UbiH was first reported to encode 2-octaprenyl-6-methoxyphenol hydroxylase in bacteria, which participates in the hydroxylation of C1 in *E. coli* CoQ8 biosynthesis. Nakahigkgsi et al. isolated and cloned the *visB* gene from *E. coli* K-12. Mutations in this gene induce cell death upon exposure to visible light. The mutated gene in the cloned strain was named *visB*, and its encoded protein is similar to the flavin monooxygenase UbiH found in *Pseudomonas fluorescens*. This implies that *visB* may be the same as *ubiH*, which is essential for CoQ production. The increased photosensitivity of the mutant strain may have resulted from the accumulation of catalytic substrates owing to the *visB* (*ubiH*) mutation, subsequently leading to the generation of reactive oxygen species (ROS) and consequent cell death [13]. Deleting *UbiH* enhanced *E. coli* resistance to D-cycloserine and increased ROS levels, indicating the antioxidative function of UbiH [14,15]. Research on the function of UbiH in biological organisms is limited, and extensive studies are being conducted to determine the function of its homologous proteins. In *E. coli*, the absence of the allelic acid gene related to *UbiH* results in the deactivation of aldehyde dehydrogenase (ALDH). This deactivation may be attributed to the accumulation of intermediates in CoQ synthesis caused by the absence of the *acd* gene, consequently affecting CoQ biosynthesis and leading to the inhibition of ALDH activity [16]. Kwon et al. showed that a UbiF-deficient mutant strain was incapable of proliferation or producing CoQ in a culture medium that solely relied on succinate as its carbon source. However, restoration of the succinate utilization and CoQ synthesis abilities of the mutant occurred through complementation with *UbiH*, suggesting a functional similarity between *UbiF* and *UbiH* [17]. In *E. coli*, the deficiency of UbiF, a protein involved in energy metabolism, increased sensitivity to diverse antibiotics and environmental stressors such as oxidative stress, acidic pH, and weak acids [18]. According to the functional characteristics of the UbiH homologous protein, we postulate that UbiH may be involved in the regulation of both ALDH activity and the stress response.

Our previous study of ALDH function in *F. graminearum* showed that the expression of *FGSG_11100*, containing the conserved UbiH domain, was downregulated in *ALDH* knockout mutants under citral stress, demonstrating the involvement of UbiH in the stress response [19]. However, its biological function in *F. graminearum* remains unclear. FgUbiH was identified as the protein encoded by *FGSG_11100* in this study. This study aimed to investigate the influence of FgUbiH on mycelial growth, conidia production, deoxynivalenol (DON) biosynthesis, and pigment synthesis and explore its role in mitochondrial function and antioxidant activity. Our results enhance the understanding of the function of UbiH in *F. graminearum*, shedding light on its possible involvement in stress response and metabolic regulation. This is crucial for identifying novel drug targets, devising effective strategies to combat FHB in wheat, and controlling DON contamination.

## 2. Materials and Methods

### 2.1. Strain Culture and Growth Conditions

A comprehensive list of all *F. graminearum* strains and plasmids used is provided in Table S1. The strains were stored at −80 °C in conidial suspensions in a carboxymethylcellulose sodium medium (CMC). To activate the strains, they were cultured on a complete medium (CM) at 25 °C for 96 h. The mycelial growth of the strains was assessed by growing them on various media, including CM, minimal media (MM), potato dextrose agar medium (PDA), and starch yeast medium (SYM) at 28 °C for 72 h [20]. We adopted the assessment method for conidium production and conidial germination, as described by Tang et al. [19]. Germination rate was calculated by observing 100 conidia at 0, 2, 4, 6, 8, and 24 h. The assessment of sexual reproduction was assayed using a carrot-infused agar growth medium, adhering to formerly established protocols [21]. In the pigment assay, the mycelia of PH-1 and the *FgUbiH* mutant were placed on a PDA solid medium and cultivated at 28 °C in darkness for 72 h. They were inoculated into a PDA liquid medium and agitated continuously at a rate of 150 rpm for 72 h to assess pigment production. Three independent repetitions were carried out for each experiment. Table S2 provides the components of all the media used in this study. 

### 2.2. Identification of FgUbiH and FgUbiH Mutant Construction

To retrieve the amino acid and nucleotide sequences corresponding to the *FGSG_11100* gene, we queried the National Center for Biotechnology Information’s (NCBI) gene database (https://www.ncbi.nlm.nih.gov/gene (accessed on 27 July 2024)). The NCBI conserved domain database was used to predict the conserved domains (https://www.ncbi.nlm.nih.gov/Structure/cdd/wrpsb.cgi (accessed on 17 May 2023)). This led to the identification of the *FGSG_11100* genetic locus that encodes homologs of the UbiH protein in *F. graminearum*, designated as FgUbiH. We conducted a BLASTP search using the FgUbiH protein sequence as a query to confirm homologous proteins in the protein databases of other species. The phylogenetic evolutionary tree for the UbiH protein was constructed using the software tool MEGA6.0 [22].

To construct the knockout mutant, we adopted the method described by Tang et al. [19], with the primers used to amplify the flanking sequences of *FgUbiH* genes (Table S3). The primers KFg1100-1F/2R and KFg1100-3F/4R were used to amplify homologous sequences measuring 786 bp and 811 bp, respectively. The hygromycin phosphotransferase (*hph*) resistance gene, amplified from plasmid pCX62, was used as a selection marker. The *hph* marker and the homologous sequences were connected using a split-marker strategy as outlined by Catlett et al. [23], and the connected fragments were then transferred to wild-type PH-1 protoplasts. The process involved the preparation of protoplasts, followed by fungal transformation steps using the established protocol outlined by Hou et al. [24]. After growing the transformants, genomic DNA was extracted using a modified CTAB method as outlined in a previous study [24]. RNA extraction was carried out using an RNA Extraction Kit from Takara Bio Inc. (Dalian, China) according to the provided instructions. The identification of the transformants was performed using polymerase chain reaction (PCR). Positive gene knockout mutants were additionally validated through quantitative real-time polymerase chain reaction using the QF/QR primers. The mutant obtained by disrupting the *FgUbiH* gene was denoted ∆*FgUbiH* mutant. In the complementation assay, we amplified a 4411 bp *FgUbiH* complementation fragment containing a 1511 bp open reading frame and its native 2900 bp promoter. The pKNT plasmid was digested using HindIII and KpnI restriction enzymes (NEB, Ipswich, MA, USA), followed by purification using a FastPure Gel DNA Extraction Mini Kit (Vazyme Biotech Co., Ltd., Nanjing, China). The complementation fragment was incorporated into the linearized pKNT plasmid to construct the FgUbiH-GFP vector using an In-Fusion Cloning Kit (Vazyme Biotech Co., Ltd., Nanjing, China). The subsequent transformation and verification steps were performed according to the methods described by Tang et al. [19].

### 2.3. Pathogenicity and DON Production Analysis

The assessment of the pathogenicity levels exhibited by different strains towards wheat heads during the flowering stage was performed according to established protocols [19]. The conidial concentration of each strain was standardized to 2–5 × 10^5^ cells/mL, and 10 μL of conidial suspension was inoculated at the center of freshly harvested wheat spikelets. The control used sterile water that was injected as a reference substance. Ten spikes were used as replicates for each strain, which were cultured for seven days post-incubation at 25 °C and monitored for the development of disease symptoms.

To analyze DON production, 5 mm diameter disks were excised from the peripheries of 5-day-old colonies of the strains and placed into 100 g of sterilized wheat grains. The inoculated grains were then kept at 28 °C with intermittent shaking for a period of 21 days before DON detection, according to the method described by Li et al. [25]. Mycelial plugs derived from each strain were grown in a liquid medium for trichothecene biosynthesis induction (TBI) media. Subsequently, the cultures were incubated at 28 °C and shaken at 150 rpm for 28 days in darkness. Collection, dehydration, and weighing of mycelia were conducted using the previous method [26]. The amount of DON was standardized per g of dried mycelia. A DON detection kit based on the enzyme-linked immunosorbent assay (Huaan Magnech Bio-Tech, Beijing, China) was used to quantify DON levels in wheat grain and TBI liquid media, according to the manufacturer’s instructions. Three replicates were there for each strain.

### 2.4. Transcriptome Sequencing Analysis

The PH-1 and ∆*FgUbiH* strains were cultured in a CM liquid medium at 28 °C with shaking at 150 rpm for 72 h. Each strain was represented by three biological replicates to ensure reproducibility. Subsequently, the RNA was extracted, purified, and used for the construction of sequencing libraries. For specific operation methods, refer to Liu et al. [27]. The libraries were then subjected to sequencing on an HiSeq Illumina next-generation sequencing platform (San Diego, CA, USA) by Origingene (Shanghai, China). Following a statistical analysis of the raw sequencing data, quality filtering is conducted to obtain clean reads. These clean reads are then aligned to the *Fusarium graminearum* PH-1 reference genome, generating mapped reads for subsequent analyses such as transcriptome assembly and expression quantification. Functional annotation is carried out, and gene expression levels are assessed. A statistical analysis is performed on the gene expression data to identify differentially expressed genes (DEG) between samples. Gene annotations were derived from the NCBI and *F. graminearum* PH-1 databases (https://www.ncbi.nlm.nih.gov/datasets/taxonomy/229533/ (accessed on 15 September 2023)). Differentially expressed genes with |log2Fold change| > 1 and a *p*-value < 0.05 were considered statistically significant [27]. Enrichment analyses were performed using the Gene Ontology (GO) and Kyoto Encyclopedia of Genes and Genomes (KEGG) databases. DEGs associated with growth, development, and DON synthesis were analyzed. Specific DEGs were confirmed using a fluorescence quantitative PCR analysis (qRT-PCR).

### 2.5. Analysis of Relative Expression of Genes

After cultivation in liquid PDA, TBI, or CM, RNA was extracted from the mycelia of PH-1 and mutant strains using an RNAiso kit (Takara, Beijing, China) according to the manufacturer’s instructions. The culture conditions of mycelium were the same as those described in Section 2.1, Section 2.3, and Section 2.4, respectively. cDNA was generated using the cDNA Synthesis Kit (Vazyme Biotech Co., Ltd., Nanjing, China). Quantification of gene expression levels was achieved through qRT-PCR using an SYBR^®^ Premix Ex TaqTM kit (Takara, Beijing, China) and specific primers (Table S3). The endogenous reference for the gene expression analysis in *F. graminearum* was the β-tubulin gene. The 2^−∆∆Ct^ method was used to quantify the gene expression levels [28]. Three replicates of qRT-PCR assays were performed.

### 2.6. Analysis of Antioxidant Function

To assess sensitivity to oxidative stress, 5 mm-diameter mycelial plugs from the peripheries of 5-day-old colonies of each strain were placed onto CM agar with and without the supplementation of 0.05% H_2_O_2_, 2.5% C_2_H_5_OH, and 0.1% CH_3_CHO. The plates were incubated at 28 °C in darkness for 72 h before the colony diameters of all strains were determined through the crisscross technique [29]. Colony morphology was documented using a camera (Nikon, Tokyo, Japan), and experiments were performed in triplicate.

To assess the antioxidant function of FgUbiH, PH-1, and mutant strains were cultivated in a CM liquid medium. After harvesting the mycelia, the activities of catalase (CAT), peroxidase (POD), and superoxide dismutase (SOD) were determined using the corresponding enzyme activity assay kits (Solarbio, Beijing, China), according to the manufacturer’s instructions. The level of H_2_O_2_ was measured using test kits provided by Solarbio, according to the manufacturer’s instructions. The DCFH-DA method was used to quantify the ROS content in accordance with the ROS assay kit (Beyotime, Shanghai, China) instructions. ROS accumulation in hyphal cells was visualized using a confocal laser scanning microscope (FV3000, OLYMPUS, Tokyo, Japan).

### 2.7. Analysis of Influence on Mitochondrial Function

To analyze the impact of FgUbiH on mitochondrial function, the activities of mitochondrial dehydrogenase and succinate dehydrogenase (SDH), mitochondrial membrane potential (MMP), and adenosine triphosphate (ATP) levels were measured. The method for mycelium preparation was the same as that described in Section 2.6 for assessing antioxidant enzyme activity. Mitochondrial dehydrogenase was detected using the 2,3-bis(2-methoxy-4-nitro-5-sulfophenyl)-5-[(phenylamino) carbonyl]-2H-tetrazolium hydroxide (XTT) method as described by Zhang et al. [30]. ATP content and SDH activity were detected using kits sourced from Solarbio (Beijing, China). The activity of ALDH was determined by using a suitable kit (Solarbio, Beijing, China). MMP was evaluated using the JC-1 dye-based MMP Assay Kit (Beyotime, Shanghai, China). Mycelia were collected and washed two times in 0.01 M phosphate-buffered saline pH 7.4 before incubating with 10 μg/mL JC-1 at 37 °C for 1 h. The cells were then washed two times with the designated JC-1 staining buffer. Images were captured using a confocal laser scanning microscope (FV3000, OLYMPUS), and alterations in cell membrane potential were identified by the conversion of JC-1 fluorescence from red to green. Carbocyanide 3-chlorophenzide was used as the positive control [31]. These experiments were repeated three times for each strain.

### 2.8. Statistical Analysis

A statistical analysis was performed using SPSS 20 (IBM Corp., Armonk, NY, USA). Significance was assessed using a one-way analysis of variance and Fisher’s least significant difference test. Graphical representations were generated using GraphPad Prism 8.0.1 (GraphPad Software, Boston, MA, USA).

## 3. Results

### 3.1. Phylogenetic Analysis of FgUbiH

**Figure 1:**
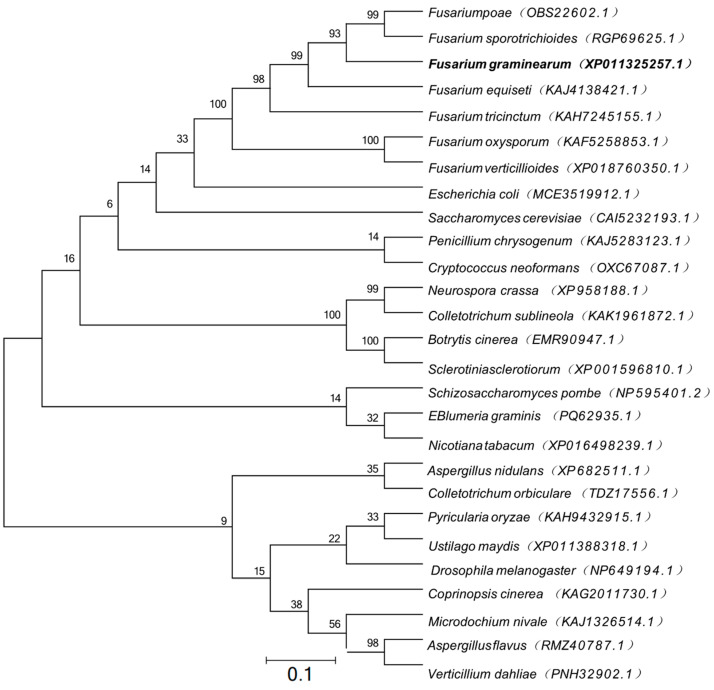
Phylogenetic analysis of FgUbiH. Phylogenetic analysis of full-length amino acid sequences of FgUbiH and its orthologs from *Fusarium poae*, *Fusarium sporotrichioides*, *Fusarium graminearum*, *Fusarium equiseti*, *Fusarium tricinctum*, *Fusarium oxysporum*, *Fusarium verticillioides*, *Escherichia coli*, *Saccharomyces cerevisiae*, *Penicillium chrysogenum*, *Cryptococcus neoformans*, *Neurospora crassa*, *Colletotrichum sublineola*, *Botrytis cinerea*, *Sclerotinia sclerotiorum*, *Schizosaccharomyces pombe*, *EBlumeria graminis*, *Nicotiana tabacum*, *Aspergillus nidulans*, *Colletotrichum orbiculare*, *Pyricularia oryzae*, *Ustilago maydis*, *Drosophila melanogaster*, *Coprinopsis cinerea*, *Microdochium nivale*, *Aspergillus flavus*, *Verticillium dahliae*. The phylogenetic tree was constructed using the neighbor-joining method with MEGA6 software. The bootstrap values displayed were calculated from 1000 replications. The presence of FgUbiH in *F. graminearum* is indicated by the black bold font.

In the genome of the sequenced *F. graminearum* strain PH-1, the gene *FGSG_11100* was predicted to encode a UbiH domain protein, which shares 22.03% amino acid identity with *Saccharomyces cerevisiae* S288C kynurenine 3-monooxygenase and 22.57% identity with *E. coli* UbiH. Therefore, the gene was named FgUbiH, a 1511 bp gene with a coding sequence of 1281 bp encoding 426 amino acids. Using BLASTP with the FgUbiH protein sequence as an input query, twenty-seven homologous proteins were identified in other species. The phylogenetic analysis indicated that seven proteins belonging to the *Fusarium* genus were grouped in a single branch, whereas UbiH proteins from other species were distributed across distinct branches (Figure 1). The results showed that FgUbiH is highly conserved within the *Fusarium* genus, suggesting its potential functional similarity.

### 3.2. FgUbiH Is Crucial for F. graminearum Vegetative Growth

**Figure 2:**
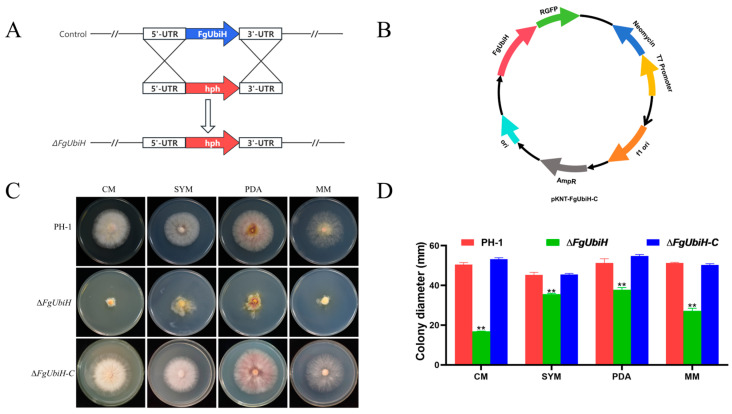
Construction of *FgUbiH* mutants and the impact of FgUbiH on vegetative growth. (**A**) *FgUbiH* deletion mutant construction. (**B**) The plasmid pKNT-FgUbiH-C was used to construct the *FgUbiH* complementation strain. (**C**) Wild-type PH-1, *FgUbiH* deletion mutant (∆*FgUbiH*), and *FgUbiH* complemented strains (∆*FgUbiH-C*) were grown on complete medium (CM), starch yeast medium (SYM), potato dextrose agar medium (PDA), and minimal media (MM) at 28 °C for 72 h. (**D**) The colony diameter of each strain. Significance was marked using “**” (*p* < 0.01).

To explore the function of FgUbiH in *F. graminearum*, *FgUbiH* deletion and complementation strains were constructed (Figure 2A,B), and their effects on the mycelial growth and morphology of the colony were investigated. The ∆*FgUbiH* mutant exhibited significantly diminished vegetative and aerial hyphal growth compared to the PH-1 and ∆*FgUbiH-C* mutant strains on both CM and MM. Additionally, the ∆*FgUbiH* mutant showed an irregular morphology (Figure 2C,D). These findings indicate the importance of FgUbiH in *F. graminearum* nutrition and polarized growth.

### 3.3. FgUbiH Is Essential for Conidiogenesis and Sexual Reproduction in F. graminearum

**Figure 3:**
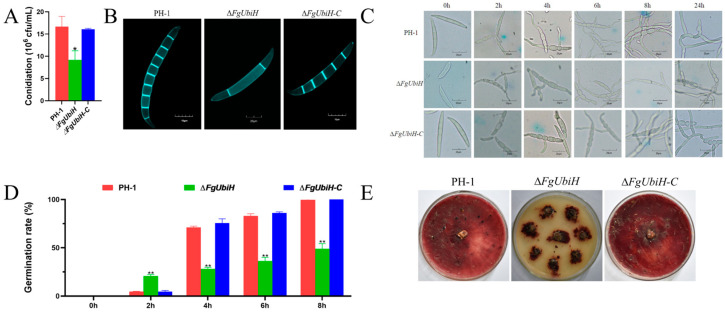
FgUbiH influences conidial production and asexual development. (**A**) Conidia production of wild-type PH-1, ∆*FgUbiH*, and ∆*FgUbiH-C* strains in CMC liquid medium for 72 h. (**B**) Conidial morphology of the wild-type PH-1, ∆*FgUbiH* and ∆*FgUbiH-C* strains cultured in CMC liquid medium for 72 h. (**C**) Conidial germination of wild-type PH-1, ∆*FgUbiH*, and ∆*FgUbiH-C* strains at different periods. (**D**) Conidia of each strain were inoculated into the CM liquid medium, and germination rate of conidia was examined under a microscope every 2 h. (**E**) Images of the perithecia produced by the PH-1, ∆*FgUbiH*, and ∆*FgUbiH-C* strains after 21 days of inoculation on carrot agar plates. Significance was marked using “*” (*p* < 0.05) and “**” (*p* < 0.01).

The ascospores and non-sexual conidia serve as crucial inocula for infecting flowering wheat heads in *F. graminearum.* A decrease in conidiation in ∆*FgUbiH* was observed than that of the wild-type and ∆*FgUbiH-C* strains by 45.0% and 42.9%, respectively (Figure 3A). The ∆*FgUbiH* mutant displayed a spore morphology that closely resembled that of the wild-type; however, there was a significant reduction in septum formation, with the majority having 2–3 septa (Figure 3B). When compared with the wild-type PH-1 strain, a significant decrease in conidia germination was observed in the ∆*FgUbiH* mutant after 4 h (Figure 3C,D). To explore the function of FgUbiH in fungal sexual reproduction, the strains were cultivated in carrot agar medium to observe perithecia development. In comparison to the PH-1 and ∆*FgUbiH-C* strains, the absence of *FgUbiH* in *F. graminearum* led to a significant decrease in perithecium formation (Figure 3E). These results indicate that FgUbiH exerts a pivotal influence on vital reproductive functions within *F. graminearum*.

### 3.4. FgUbiH Plays a Crucial Role in Both Pathogenicity and the Production of DON

**Figure 4:**
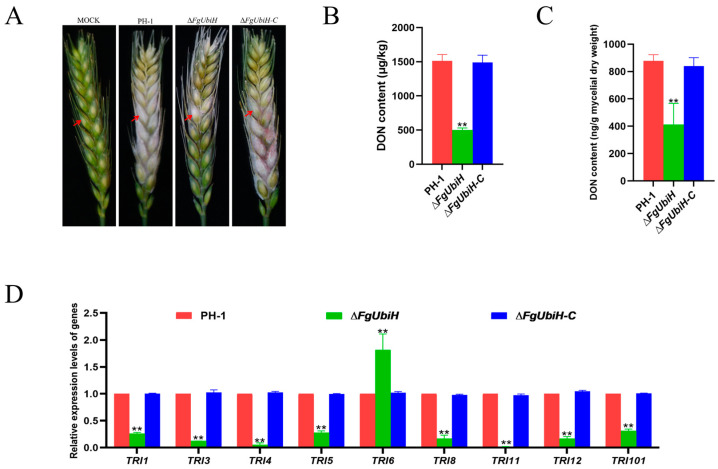
FgUbiH is essential for virulence display. (**A**) The pathogenicity of ∆*FgUbiH* mutant on flowering wheat heads showed a significant decrease compared with the wild-type PH-1 and ∆*FgUbiH-C* strains. Conidia suspensions from different strains were inoculated into flowering wheat heads in humid conditions, and disease symptoms were assessed after 7 days. The red arrow indicates the location for inoculating the spore suspension of the strain. (**B**) A decrease in deoxynivalenol (DON) content was observed in the ∆*FgUbiH* mutant strain. PH-1, ∆*FgUbiH,* and ∆*FgUbiH*-C strains were incubated in wheat grain for 21 days. (**C**) A reduction in DON content was noted in the ∆*FgUbiH* mutant strain. All of the strains were incubated in a trichothecene biosynthesis induction (TBI) liquid medium for 28 days. Drying and weighing were conducted on the mycelia of each strain to determine the fungal biomass. (**D**) In the ∆*FgUbiH* mutant, the relative expression levels of several TRI genes involved in trichothecene biosynthesis were notably reduced, except for TRI6. Significance was marked using “**” (*p* < 0.01).

To examine the functions of FgUbiH in the pathogenicity of *F. graminearum* in host plants, infection assays were performed on wheat heads during their flowering stage. The wild-type and ∆*FgUbiH-C* strains both showed pronounced symptoms of FHB, whereas the ∆*FgUbiH* mutant exhibited significantly reduced infection capacity (Figure 4A). To determine the importance of FgUbiH in the regulation of DON synthesis, the mycelium of the strains was incubated with wheat grain and TBI medium for 21 and 28 days, respectively, and the DON production levels were examined. A significant reduction in DON content was observed in the ∆*FgUbiH* mutant (Figure 4B,C), consistent with the findings of our pathogenicity tests. Subsequently, the relative expression of TRI genes (*TRI1*, *TRI3*, *TRI4*, *TRI5*, *TRI6*, *TRI8*, *TRI11*, *TRI12*, and *TRI101*) was assessed in PH-1 and *FgUbiH* mutant strains. The results demonstrated significant downregulation in the transcription levels of TRI genes, except for *TRI6*, in the ∆*FgUbiH* mutant (Figure 4D). These results demonstrate the regulatory function of FgUbiH in pathogenicity and DON synthesis.

### 3.5. Transcriptional Profiling in Wild-Type and ∆FgUbiH Strains

**Figure 5:**
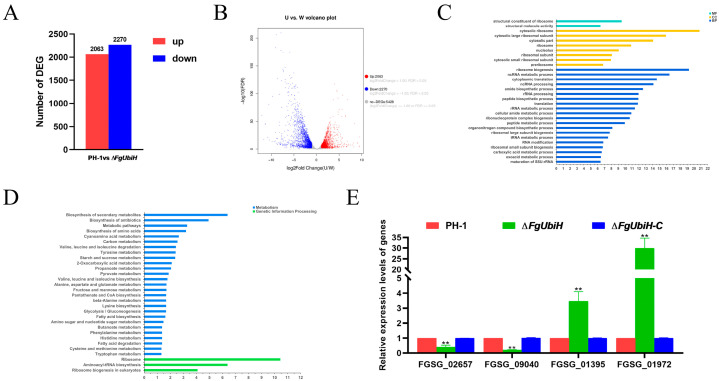
Transcriptomic analysis of ∆*FgUbiH* strain compared with wild-type PH-1 strain. (**A**) The up and down-regulated differentially expressed genes (DEGs) in the ∆*FgUbiH* mutant strain compared with the wild-type PH-1 strain. (**B**) Volcano plots of DEGs. W: wild-type PH-1 strain; U: ∆*FgUbiH* mutant strain. (**C**) Gene Ontology enrichment analysis of DEGs (Padj < 0.05). (**D**) Kyoto Encyclopedia of Genes and Genomes analysis of DEGs. (**E**) Relative expression levels of genes related with tryptophan metabolism in wild-type PH-1 and *FgUbiH* mutant. RNA was extracted from the mycelia of each strain following a 3-day incubation in a CM. Significance was marked using “**” (*p* < 0.01).

To elucidate the regulatory role of FgUbiH in controlling mycelial growth and DON biosynthesis in *F. graminearum*, RNA-seq was conducted to compare the transcriptional profiles of PH-1 with those of the ∆*FgUbiH* mutant strain. In ∆*FgUbiH*, 4333 DEGs were identified, with 2063 (47.61%) upregulated and 2270 (52.39%) downregulated compared with the PH-1 strain (Figure 5A), suggesting that FgUbiH affects the transcription of various genes. The volcano plot displayed a roughly symmetrical distribution of DEGs (Figure 5B), demonstrating the reliability of the transcriptome data.

Figure 5C displays the top 30 GO terms of the DEGs in the ∆*FgUbiH* strain. The genes that were most prominently enriched in the molecular function group were ribosomal and molecular structure activities. In the cellular component, DEGs were mostly enriched in the cytosolic ribosome, cytosolic large ribosomal subunit, and cytosolic part. In biological processes, the DEGs were mainly enriched in ribosome biogenesis, ncRNA metabolic processes, and cytoplasmic translation. These findings suggested that the majority of DEGs were associated with oxidative stress.

Figure 5D displays the top 30 pathways from the KEGG pathway analysis of DEGs in the ∆*FgUbiH* strain. A significant number of DEGs were enriched in the biosynthesis of secondary metabolites, biosynthesis of antibiotics, amino acid metabolism, and ribosomes. DEGs regulated by FgUbiH were identified to explore the mechanisms by which FgUbiH regulates growth, development, and secondary metabolites (Table 1). To confirm the transcriptome data, the relative expression of tryptophan metabolism-related genes was analyzed. The ∆*FgUbiH* strain exhibited downregulation of *FGSG_02657* and *FGSG_09040* expression, whereas *FGSG_01395* and *FGSG_01972* expression was upregulated (Figure 5E). These findings were consistent with the results from the RNA-seq analysis.

### 3.6. FgUbiH Regulates the Production of Pigment

**Figure 6:**
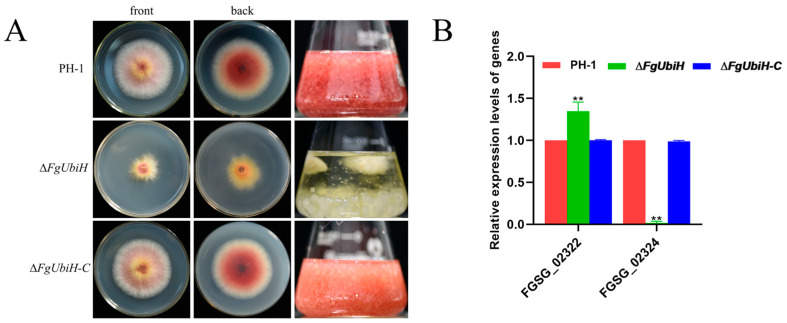
The impact of FgUbiH on pigment formation. (**A**) The pigment changes of wild-type, ∆*FgUbiH*, and ∆*FgUbiH-C* strains were observed after 72 h of inoculation on PDA plates and in a PDA liquid medium, respectively. (**B**) Relative expression levels of genes related to pigment synthesis in wild-type PH-1 and *FgUbiH* mutants were measured. RNA was extracted from the mycelia of each strain after a 3-day incubation in a PDA medium. Significance was marked using “**” (*p* < 0.01).

Transcriptome sequencing revealed significant changes in DEG expression linked to pigment synthesis in the ∆*FgUbiH* strain. Therefore, the PH-1 and ∆*FgUbiH* strains were cultivated separately on solid PDA plates and in a liquid medium. The wild-type strain produced red pigment, whereas the deletion mutant strain did not exhibit red pigment production (Figure 6A), indicating the critical role of FgUbiH in pigment biosynthesis. The relative expressions of *PKS12* (FGSG_02324) and *AURJ* (FGSG_02322) were quantified. The results showed a significant downregulation of PKS12 expression and an upregulation of *AURJ* in the ∆*FgUbiH* mutant (Figure 6B), suggesting that FgUbiH regulates pigment production by influencing the expression of genes involved in pigment synthesis.

### 3.7. FgUbiH Is Involved in the Antioxidant Function of F. graminearum

**Figure 7:**
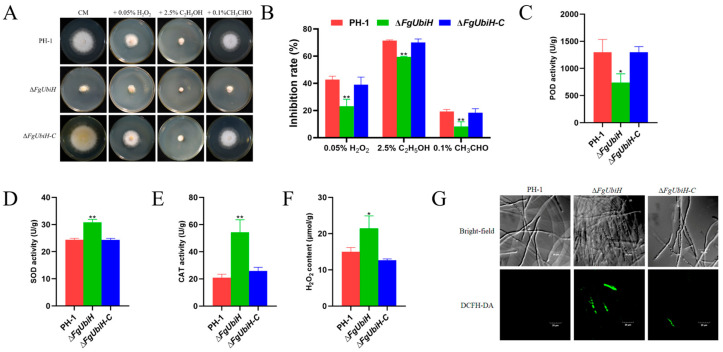
Effects of *FgUbiH* deletion on antioxidant function in *F. graminearum*. (**A**) Wild-type PH-1, ∆*FgUbiH*, and ∆*FgUbiH-C* mutants grown on CM plates with or without 0.05% H_2_O_2_, 2.5% C_2_H_5_OH, and 0.1% CH_3_CHO for 3 days. (**B**) Growth inhibition rate of oxidative stress factors to each strain. (**C**) The enzyme activity of peroxidase (POD). (**D**) The enzyme activity of superoxide dismutase (SOD). (**E**) The enzyme activity of catalase (CAT). (**F**) The concentration of H_2_O_2_. (**G**) Reactive oxygen species (ROS) accumulation was observed using laser confocal scanning microscopy (LSCM) (FV3000, OLYMPUS Corporation, Tokyo, Japan). The wild-type PH-1, ∆*FgUbiH*, and ∆*FgUbiH-C* mutant strains were grown on CM medium at 28 °C for 72 h. The significance level was tested by unpaired *t*-test (* *p* < 0.05, ** *p* < 0.01).

To evaluate the influence of the FgUbiH deletion on cellular antioxidant characteristics in *F. graminearum*, the oxidative stress response, antioxidant enzyme activity, and intracellular ROS levels were evaluated. In the oxidative stress reaction, the sensitivity of the ∆*FgUbiH* strain to 0.05% H_2_O_2_, 2.5% C_2_H_5_OH, and 0.1% CH_3_CHO was significantly reduced compared to the wild-type strain (Figure 7A,B). Compared with the wild-type strain, the RNA-seq analysis showed that two POD genes, *FGSG_00308* and *FGSG_00407*, were significantly downregulated in the ∆*FgUbiH* strain, and one CAT gene, *FGSG_05685*, was significantly upregulated (Table 1). POD activity decreased by 43.1%, whereas SOD and CAT activities significantly increased by 26.5% and 158.4%, respectively (Figure 7C–E). ROS levels were determined by indirect detection of H_2_O_2_ concentrations and direct ROS measurement in the *FgUbiH* mutant strain. A significant increase in H_2_O_2_ concentrations was detected in the ∆*FgUbiH* strain (Figure 7F). Similarly, fluorescence analysis demonstrated heightened ROS levels in the ∆*FgUbiH* strain (Figure 7G), which correlated with the reduced expression of antioxidant genes and antioxidant enzyme activities. These findings indicate that the absence of *FgUbiH* impairs the antioxidant activity. FgUbiH potentially modulates growth development in *F. graminearum* by regulating antioxidant levels.

### 3.8. FgUbiH Influences Mitochondrial Function

**Figure 8:**
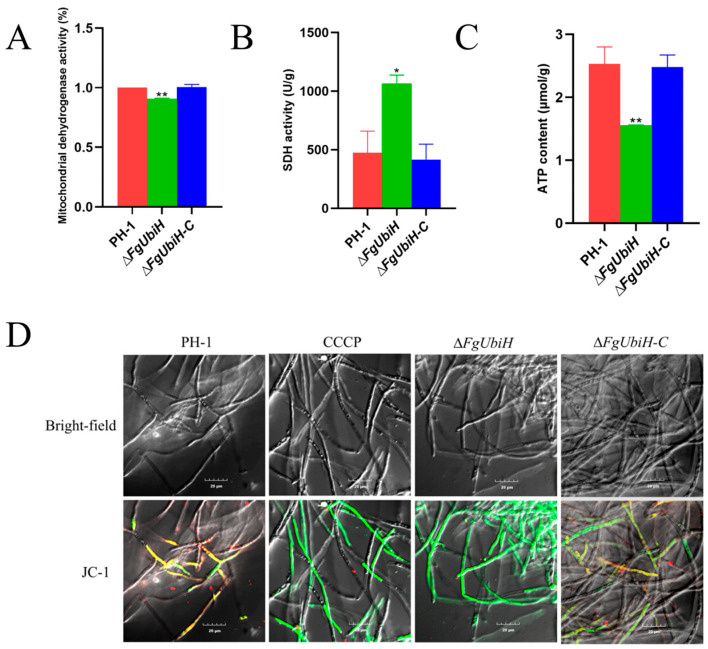
Effects of *FgUbiH* deletion on mitochondrial function. (**A**) Enzyme activity of mitochondrial dehydrogenase. (**B**) Enzyme activity of succinate dehydrogenase (SDH). (**C**) ATP content. (**D**) Fluorescence images of JC-1 staining of PH-1, ∆*FgUbiH*, and ∆*FgUbiH-C* mycelia were observed using LSCM. A high ratio of Red fluorescence was observed, indicating the formation of J-aggregates in the mitochondrial matrix and a high mitochondrial membrane potential (MMP). The presence of a high level of green fluorescence indicated that JC-1 was in a monomeric state, signifying a low MMP. The yellow fluorescence indicated that some JC-1 probes are in an intermediate state, suggesting that the changes in MMP were not significant. The wild-type PH-1 strain, ∆*FgUbiH*, and ∆*FgUbiH-C* mutant strains were cultivated in CM for 72 h, following which mycelia samples were harvested for analysis. The significance level was tested by unpaired *t*-test (* *p* < 0.05, ** *p* < 0.01).

To elucidate the influence of the *FgUbiH* deletion on mitochondrial function, we examined the activities of mitochondrial dehydrogenases and SDH, ATP levels, and alterations in MMP. The *FgUbiH* deletion mutant exhibited a significant decline in mitochondrial dehydrogenase activity and a significant increase in SDH activity compared to the wild-type (Figure 8A,B). ATP content in the PH-1 strain was 2.53 μmol/g, whereas in the ∆*FgUbiH* mutant strain, it showed a significant decrease to 1.55 μmol/g (Figure 8C). MMP measurements showed that the hyphae of the PH-1 strain displayed a higher intensity of red fluorescence than green fluorescence, whereas the Δ*FgUbiH* strain exhibited significant green fluorescence and only a faint red fluorescence (Figure 8D). The findings suggest that FgUbiH is crucial in preserving mitochondrial function.

### 3.9. FgUbiH Participates in CoQ Synthesis and Regulates Acetaldehyde Dehydrogenase Activity

**Figure 9:**
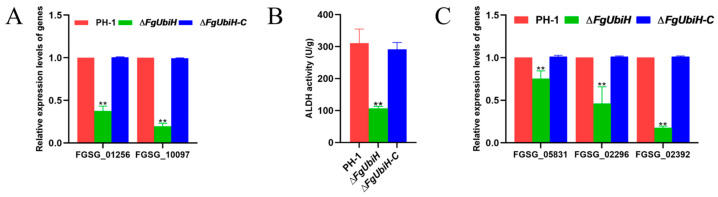
The impact of FgUbiH on coenzyme Q synthesis and aldehyde dehydrogenase (ALDH) activity. (**A**) The relative expression levels of genes involved in terpene skeleton synthesis in wild-type strains and *FgUbiH* mutant strains. (**B**) The activity of acetaldehyde dehydrogenase in wild-type and *FgUbiH* mutant strains. (**C**) The relative expression levels of ALDH genes in wild-type and *FgUbiH* mutant strains. The wild-type PH-1 strain, ∆*FgUbiH*, and ∆*FgUbiH-C* mutant strains were cultivated in CM liquid medium at 28 °C and 150 rpm for 72 h, following which mycelia samples were harvested for analysis. The significance level was tested by unpaired *t*-test (** *p* < 0.01).

Synthesis of the terpenoid backbone is a prerequisite for the synthesis of CoQ. RNA-seq analysis showed significant downregulation of genes involved in the terpene skeleton biosynthetic pathway in the *∆FgUbiH* strain, which was validated using qRT-PCR, showing the downregulation of undecapenyl diphosphate synthase (*FGSG_01256*) and polyisoprene synthase (*FGSG_10097*) (Figure 9A). This suggests that FgUbiH is crucial in CoQ synthesis through the regulation of the genes responsible for terpene backbone biosynthesis. Additionally, FgUbiH regulates the activity of acetaldehyde dehydrogenase. In the ∆*FgUbiH* mutant strain, there was a significant reduction in aldehyde dehydrogenase activity (Figure 9B) and a distinct downregulation in the expression of three ALDH genes (*FGSG_05831*, *FGSG_02296*, and *FGSG_02392*) (Figure 9C). This suggests that FgUbiH impacts the regulation of acetaldehyde detoxification in *F. graminearum*. These findings suggest that FgUbiH participates in the synthesis of CoQ and regulates ethanol metabolism by controlling the activity of ALDH.

**Figure 10:**
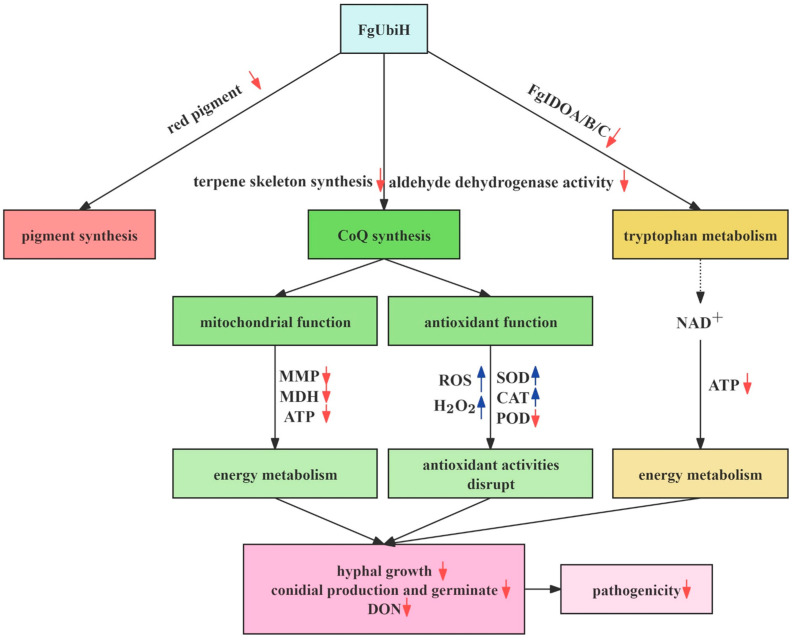
Proposed model for the role FgUbiH in *Fusarium graminearum*. The red arrow indicates downregulation in ΔFgUbiH strain compared to control, while the blue arrow signifies upregulation in certain parameters.

Taken together, our findings highlight the significant role of FgUbiH in *F. graminearum* (Figure 10). FgUbiH influences pigment synthesis, coenzyme Q production, and tryptophan metabolism in *F.graminearum*. The deletion of *FgUbiH* disrupts mitochondrial function and antioxidant capability, which consequently affects energy metabolism and antioxidant enzyme activity in *F. graminearum*. As a result, mycelial growth is slowed, spore production is reduced, deoxynivalenol (DON) toxin production is diminished, and pathogenicity is weakened.

## 4. Discussion

CoQ is vital for electron transfer [32]. Previous studies have reported that CoQ plays essential roles in oxidative phosphorylation, anti-oxidation, and diverse mitochondrial processes [33,34,35]. Despite the conservation of CoQ across different species, the extent of diversity in CoQ biosynthesis pathways has not been extensively investigated. The majority of knowledge regarding eukaryotic CoQ biosynthesis is based on studies conducted in yeast and bacteria [34], and the CoQ biosynthesis pathway and its associated genes in filamentous fungi remain incompletely understood. In this study, we identified the biological function of FgUbiH, a protein involved in CoQ biosynthesis, and provided insights into its role in growth regulation, toxin production, antioxidant activity, and mitochondrial function in *F. graminearum*. We established a molecular model for studying UbiH proteins in filamentous fungi and provided a foundation for the development of molecular strategies to prevent and control crop diseases caused by pathogenic fungi.

Previous studies have reported that UbiH is essential for CoQ biosynthesis in certain bacteria [36]. Deletion of *UbiH* in *E. coli* leads to light sensitivity and inhibition of cell growth. The absence of UbiH was considered the cause of this phenomenon, leading to an excess of substrates that interfere with CoQ biosynthesis. Accumulated intermediates generated ROS upon light exposure, causing cell death [13]. Additionally, Jiang et al. attempted to disrupt UbiH in *P. aeruginosa* M18 but failed to isolate mutant strains. This suggests that the absence of UbiH may hinder CoQ9 biosynthesis, resulting in bacterial death [37]. Moreover, studies have shown that a mutant strain of *E. coli* lacking UbiF could not grow on succinic acid as the sole carbon source. Complementation of the mutant with UbiH restored growth, indicating that UbiH and UbiF proteins are essential for cell growth [17]. These studies highlight the significant role of UbiH in cellular growth processes within biological organisms. Consistent with these findings, our results demonstrated that deletion of *FgUbiH* hindered hyphal growth, conidiation, and sexual spore formation, indicating the regulatory role of FgUbiH during the growth and progression. FgUbiH is involved in CoQ synthesis. CoQ is a pivotal element within the cellular respiration chain and performs an indispensable function in energy metabolism [3]. The absence of FgUbiH can impair CoQ synthesis, hampering the efficiency of energy metabolism. This deficiency in energy supply may retard the growth rate of the colonies. Transcriptional analysis of the *FgUbiH*-deletion mutant confirmed this finding and GO enrichment analysis revealed a significant downregulation in the expression of *FGSG_04584*, *FGSG_01351*, and *FGSG_05524,* which regulate filamentous growth. These results support the influence of FgUbiH on fungal growth and development.

Previous studies have demonstrated that CoQ is vital for the pathogenicity of various pathogens by influencing key metabolic pathways [38]. For instance, in pathogenic *Francisella novicida*, the growth of Tn-ubiCFn (a transposon mutation of the *UbiC* gene) was severely impaired when cultured under aerobic conditions in Chamberlain media, and a significant reduction in CoQ8 content was observed. CoQ8 deficiency hindered the pathogenicity of *F. novicida* against *Galleria mellonella*. These results highlight the significance of CoQ in the *Francisella* life cycle and its contribution to pathogenicity [38]. In our study, the disruption of FgUbiH impaired the ability of the pathogen to colonize wheat heads and significantly reduced DON production. These effects are consistent with the observed downregulation of *TRI* genes in the ∆*FgUbiH* mutant. This indicates that FgUbiH exerts a positive regulatory effect on DON biosynthesis by modulating *TRI* gene expression. DON is a well-studied mycotoxin and virulence determinant that is critical in the disease-causing capability of *F. graminearum* [39]. We hypothesized that the absence of FgUbiH would lead to a decrease in DON synthesis, thereby diminishing pathogenicity. Additionally, this study identified a significant downregulation of tryptophan metabolism-related genes, including *FgIDOA*, *FgIDOB*, and *FgIDOC*, in the ∆*FgUbiH* mutant (Table 1). A previous study demonstrated that these genes are found in *F. graminearum* and encode three homologous enzymes responsible for catalyzing the initial step in the decomposition of tryptophan, thereby supplying a precursor for the synthesis of the redox coenzyme NAD^+^ in fungal cells [40]. Deletion of *FgIDOA* affects growth, pathogenicity, and DON production owing to insufficient NAD^+^ levels. NAD holds a crucial function in both energy metabolism and antioxidant processes [41]. The significant downregulation of the *FgIDO* genes in the ∆*FgUbiH* mutant suggests that UbiH may regulate the expression of these genes. Since DON production is affected by the absence of *FgIDOA* and the expression of *FgIDO* genes decreases in the ∆*FgUbiH* mutant, it can be inferred that FgUbiH may have an indirect impact on DON production. Reduced levels of NAD^+^ owing to the downregulation of FgIDO could affect DON synthesis. This underscores the importance of FgUbiH in normal physiological functions and fungal toxicity. These results demonstrate that FgUbiH has a pivotal function in the virulence and regulation of DON biosynthesis. Our results are consistent with previous findings that have highlighted the importance of CoQ in *F. novicida* virulence [38].

PKS12 and AURJ are regulators of the pigment-producing biosynthetic pathway in *F. graminearum*. Knockout of the *PKS12* gene caused a significant decrease in the red pigment in the colonies and the emergence of an albino phenotype [42]. As a coregulatory factor of the *PKS12* gene cluster, Aur modulates the production of red and yellow pigments in *Fusarium* spp. A study by Westphal et al. showed that deletion of aurR2 altered the ratio of rubrofusarin to aurofusarin, and upregulation of Aur resulted in an increase in aurofusarin. Two genes, *PKS12* (FGSG_02324) and *AURJ* (FGSG_02322), govern the biosynthetic pathway of pigments in *F. graminearum* [43]. In our study, the *FgUbiH* deletion mutant exhibited a loss of red pigment in liquid PDA culture, accompanied by a significant increase in aurofusarin content. The transcriptome analysis revealed a significant downregulation of the *PKS12* gene (*FGSG_02324*) associated with the red pigment and a significant upregulation of the transcription factor *Aur* gene (*FGSG_02322*), which was validated using fluorescence quantitative PCR. These findings highlight the crucial role of FgUbiH in regulating pigment biosynthesis.

The mitochondrion functions as a key producer of ROS within cells [44], and the generation of low ROS levels can act as cell signaling to promote cellular homeostasis [3]. Excessive ROS can cause molecular harm by oxidizing lipids, proteins, and DNA, leading to cellular aging and impaired growth potential [45,46]. Various antioxidant defense systems, such as SOD, POD, and CAT, are present in all organisms to restrict and remove harmful ROS. CoQ acts as an electron carrier and concurrently functions as an antioxidant [32,47]. In our study, deletion of *FgUbiH* significantly impaired the oxidative stress response of the strain, as evidenced by its reduced sensitivity to oxidative stressors such as H_2_O_2_, CH_3_CHO, and C_2_H_5_OH. The RNA-seq analysis corroborated the phenotypic observations by revealing significant regulation of genes encoding for antioxidant enzymes in the ∆*FgUbiH* strain. The POD genes *FGSG_00308* and *FGSG_00407* were substantially downregulated, and the CAT gene *FGSG_05685* was substantially upregulated. This transcriptional repression has profound implications for the enzymatic detoxification of ROS within cells. Consistent with the gene expression data, the POD, CAT, and SOD enzymatic activities were altered in the ∆*FgUbiH* strain. A decrease in POD activity was observed, accompanied by increased CAT and SOD activities. This dichotomy in enzyme activity suggests a possible compensatory mechanism to counteract the loss of FgUbiH function; however, it appears to be insufficient to restore the normal antioxidant capacity. These findings elucidate the pivotal role of FgUbiH in the antioxidant machinery of *F. graminearum.* Biochemical assessments further confirmed the impact of *FgUbiH* deletion on cellular redox balance, with H_2_O_2_ and ROS levels significantly increased in the ∆*FgUbiH* strain. These findings are consistent with the elevated accumulation of ROS in a *UbiH*-deficient mutant of *E. coli* [11]. Excessive ROS levels can damage cells and impair their growth, potentially contributing to the slow hyphal growth observed in the *FgUbiH* deletion mutant. The accumulation of ROS is indicative of an overwhelmed antioxidant system that is unable to neutralize the oxidative burden effectively. Collectively, our findings suggest that FgUbiH exerts a pivotal influence on modulating the antioxidant responses of *F. graminearum*. This insight into the regulatory role of FgUbiH on ROS levels provides a foundation for further research on the complex interplay between antioxidant mechanisms and secondary metabolite production in fungi. Our findings suggest that by participating in CoQ biosynthesis, UbiH plays a pivotal role in sustaining cellular redox equilibrium and safeguarding cells from oxidative damage.

CoQ is vital for electron transmission in the mitochondrial respiratory chain and is closely linked to mitochondrial function [48]. CoQ serves as an essential electron transporter within the cellular respiratory chain of eukaryotic organisms, facilitating the transfer of electrons between complexes I/II and III, maintaining efficient ATP production and overall mitochondrial function [49]. Our study revealed a significant reduction in mitochondrial dehydrogenase activity and an increase in SDH activity in the ∆*FgUbiH* mutant. The ∆*FgUbiH* mutant strain exhibited a significant decrease in ATP levels. These findings are consistent with those of a previous study showing that disruptions in CoQ biosynthesis or function can impair the cellular respiratory chain, reduce ATP production, and alter the enzyme activity of various dehydrogenases [50]. For instance, studies in yeast have demonstrated that mutations affecting CoQ synthesis severely impair respiratory function and reduce cellular ATP levels [51]. Similar observations have been reported in other organisms, where CoQ deficiency leads to compromised mitochondrial function and energy metabolism [52,53,54]. Mitochondrial dehydrogenases play crucial roles in cellular respiration by catalyzing the oxidation of various substrates to produce ATP [55]. The specific activity levels of these enzymes indicate the metabolic state and health of cells. In the *FgUbiH* deletion mutant, a significant decrease in mitochondrial dehydrogenase activity suggests a reduced capacity for substrate oxidation, which could impair the ability of the cell to efficiently generate ATP. This is indicative of a potential decline in cellular energy production and may underlie the slower growth of the mutant strain. SDH is a component of the respiratory complex II that is also involved in the tricarboxylic acid cycle [56]. Its increased activity in the ∆*FgUbiH* strain might be an attempt by the cell to sustain ATP production through the tricarboxylic acid cycle, even when other dehydrogenases are less active. The reduced ATP in the ∆*FgUbiH* mutant is due to decreased mitochondrial dehydrogenase activity, highlighting their role in cellular energy balance. MMP is the electrical potential difference across the mitochondrial inner membrane and is critical for assessing mitochondrial function and cellular energy metabolism. Disruption of MMP is a vital event in programmed cell death and apoptosis [57]. In this study, MMP assessments revealed diminished red fluorescence and enhanced green fluorescence in the Δ*FgUbiH* strain, indicating disrupted membrane potential. Disruptions in MMP may result in reduced ATP synthesis, affecting cell growth and viability, which could contribute to the slow growth and decreased sporulation observed in the *FgUbiH* mutant. Collectively, our results underscore the pivotal role of FgUbiH in preserving mitochondrial function in *F. graminearum*. This gene appears to be fundamental for ensuring adequate CoQ levels and, consequently, correct functioning of the mitochondrial respiratory chain highlighting its importance in cellular energy homeostasis.

Mutations or deletions in genes associated with CoQ synthesis can lead to significant alterations in the type of CoQ or changes in physiological characteristics. For instance, studies in *E. coli* have demonstrated that mutations in *ispB*, which encodes octaprenyl diphosphate synthase, alter the production of various CoQ types and their biochemical properties [58]. Similar discoveries were reported in *S. cerevisiae*, where CoQ1 deficiency results in impaired CoQ production and reduced mitochondrial functionality [59]. Our data showed that FgUbiH is essential for regulating terpene biosynthesis genes, highlighting its crucial role in CoQ synthesis in *F. graminearum.* In addition, we identified a novel function of FgUbiH in ethanol metabolism regulation. The deletion of *FgUbiH* caused a significant decline in acetaldehyde dehydrogenase activity and a significant downregulation of ALDH genes (*FGSG_05831*, *FGSG_02296*, and *FGSG_02392*). This is consistent with studies reporting that defects in the *acd* gene (allelic to *UbiH*) result in the inactivation of acetaldehyde dehydrogenase activity in *E. coli*. Restoration of this activity occurred upon the addition of CoQ-0 to cell extracts, indicating the specific inhibition of acetaldehyde dehydrogenase activity caused by intermediates of CoQ synthesis that accumulated in the *acd* mutants [16]. Previous studies have proved that the absence of the *FGSG_05831*, encoding ALDH, resulted in decreased ALDH activity and reduced DON toxin synthesis in *F. graminearum* [19]. Similarly, in our study, the *FgUbiH* mutant with a deficiency in ALDH activity exhibited reduced DON levels. This highlights the association among CoQ synthesis, ethanol metabolism, and mycotoxin production in *F. graminearum*. Our findings highlight the important function of FgUbiH in CoQ synthesis and ethanol metabolism in *F. graminearum*, suggesting potential targets for genetic or chemical interventions to mitigate the impact of FHB.

## 5. Conclusions

Our results demonstrate that the deficiency of FgUbiH affects the biosynthesis of CoQ, diminishes antioxidant capacity, and disrupts mitochondrial function, ultimately hindering hyphal and spore production and reducing DON synthesis. Our study reveals the regulatory function of FgUbiH in CoQ biosynthesis in *F. graminearum* and highlights the primary role of the *UbiH* gene family in this fungal species. Additional research is warranted to confirm the subcellular localization of FgUbiH and its role in hydroxylation and assess how the absence of this gene impacts the types and characteristics of CoQ. Further studies are required to understand the evolution of UbiH in pathogenic fungi and its relationship with pathogenicity and toxin synthesis. The findings of this study provide a strong foundation for understanding UbiH functions in filamentous fungi, deepen our comprehension of the toxicity and pathogenic mechanisms of *F. graminearum,* and highlight the potential of targeting FgUbiH as an effective strategy to manage FHB in cereal crops.

## Figures and Tables

**Table 1:** Differentially expressed genes regulated by FgUbiH.

Gene Category	Function	Log2 (fc)	*p*-Value
Terpenoid backbone biosynthesis			
FGSG_01256	Putative undecaprenyl diphosphate synthase	−1.1187	5.59 × 10^−12^
FGSG_10097	Polyprenyl synthetase	−1.8095	3.74 × 10^−12^
Tryptophan metabolism			
FGSG_02657	Indoleamine 2,3-dioxygenase, FgIDOA	−1.4644	1.04 × 10^−13^
FGSG_04828	Indoleamine 2,3-dioxygenase, FgIDOB	−3.8635	2.03 × 10^−4^
FGSG_09040	Indoleamine 2,3-dioxygenase, FgIDOC	−7.2504	1.49 × 10^−17^
FGSG_01972	Sulfite reductase, Cytochrome P450	3.0176	6.96 × 10^−59^
FGSG_02287	Acyl-CoAoxidase	−1.3839	1.10 × 10^−10^
FGSG_02392	Aldehyde dehydrogenase family	−2.0948	2.26 × 10^−8^
FGSG_05087	Acetyl-CoA acetyltransferase	−3.0119	4.88 × 10^−51^
FGSG_07596	cytochrome P450	−1.2069	7.96 × 10^−4^
FGSG_11484	Acyl-CoA dehydrogenase	−3.7165	6.94 × 10^−7^
FGSG_13111	Enoyl-CoA hydratase, carnithine racemase	−1.3012	9.73 × 10^−9^
FGSG_04131	kynureninase	2.1986	1.76 × 10^−13^
FGSG_04829	kynureninase	−3.4876	1.54 × 10^−24^
Antioxidase genes			
FGSG_00308	peroxidase	−1.0632	2.00 × 10^−6^
FGSG_00407	peroxidase	−1.1287	4.94 × 10^−5^
FGSG_05685	catalase	1.1916	2.12 × 10^−2^
Energy metabolism			
FGSG_00429	ATP synthase E chain	−1.1627	8.34 × 10^−11^
FGSG_05266	ATP synthase subunit K	−1.0777	4.18 × 10^−9^
FGSG_05470	V-type proton ATPase 16 kDa proteolipid subunit	−2.1763	1.61 × 10^−14^
FGSG_09430	ATP synthase j chain	−1.0394	2.08 × 10^−9^
FGSG_00743	CybS, succinate dehydrogenase cytochrome B small subunit	−1.3506	4.10 × 10^−12^
Pigment synthesis genes			
FGSG_02322	Major Facilitator Superfamily, AurT	2.1693	3.98 × 10^−11^
FGSG_02323	Fungal specific transcription	1.5124	1.49 × 10^−10^
FGSG_02324	PKS12 Beta-ketoacyl synthase	−2.6789	1.70 × 10^−2^
FGSG_02749	ABC transporter	−2.7065	1.02 × 10^−32^
Aldehyde dehydrogenase genes			
FGSG_00490	Aldehyde dehydrogenase family	−2.0255	2.40 × 10^−23^
FGSG_05831	Aldehyde dehydrogenase family	−1.3580	2.92 × 10^−9^
FGSG_02296	Aldehyde dehydrogenase family	−8.0955	1.91 × 10^−12^
FGSG_02392	Aldehyde dehydrogenase family	−2.0948	2.26 × 10^−8^
Cytochrome c oxidase			
FGSG_06268	Cytochrome c oxidase subunit VIb	−1.4264	1.31 × 10^−2^
Cell growth			
FGSG_04584	Glycosyl hydrolase family 76	−7.5134	2.62 × 10^−7^
FGSG_05524	PalH/RIM21	−1.2573	8.38 × 10^−13^
FGSG_01351	Beta-glucosidase	−1.4356	5.59 × 10^−16^

## Data Availability

PacBio and Illumina Sequencing data have been submitted to the National Center for Biotechnology Information (NCBI) Sequence Read Archive (SRA) under Bio-Project: PRJNA1120214.

## Supplementary Materials

The following supporting information can be downloaded at: https://www.mdpi.com/article/10.3390/microorganisms12102093/s1, Table S1: Strains and plasmids used in this study; Table S2: Medium used in this study; Table S3: Primer sequence used in this study.
