# Supplementary material for: FgUbiH Is Essential for Vegetative Development, Energy Metabolism, and Antioxidant Activity in Fusarium graminearum
Source: Microorganisms. 2024 Oct 20;12(10):2093. doi: 10.3390/microorganisms12102093 (PMC11509934; doi:10.3390/microorganisms12102093)
Supplement: Supplementary file 1 [file microorganisms-12-02093-s001.zip › Table S1.pdf]

Table S1. Strains and plasmids used in this study.

Strains and plasmids	Description	Sources
Strains		
F.graminearum PH-1	Wild-type strain	Provided by ZongHua Wang
Δ*FgUbiH*	*F.graminearum* PH-1derivative, Δ*FgUbiH*::hygB	This study
Δ*FgUbiH-C*	*F.graminearum* PH-1derivative, Δ*FgUbiH*::hygB, FgUbiH-Com	This study
DH5α	*Escherichia coli*	Preserved in our laboratory
Plasmids		
pKNTG	For the construction of complementary mutant, G418	Provided by ZongHua Wang
pCX62	For the construction of deletion mutants, hygB	Provided by ZongHua Wang
