# Supplementary material for: FgUbiH Is Essential for Vegetative Development, Energy Metabolism, and Antioxidant Activity in Fusarium graminearum
Source: Microorganisms. 2024 Oct 20;12(10):2093. doi: 10.3390/microorganisms12102093 (PMC11509934; doi:10.3390/microorganisms12102093)
Supplement: Supplementary file 1 [file microorganisms-12-02093-s001.zip › Table S2.pdf]

Table S2. Medium used in this study.

Medium	Nutrient content
CM	yeast extract 6 g/L, casamino acid 6 g/L, sucrose 10 g/L, agar 20 g/L
CMC	sodium carboxymethyl cellulose 15 g/L, NH_4NO_3 1 g/L, KH_2PO_4 1 g/L, $MgSO_4 \cdot 7H_2O$ 0.5 g/L, yeast extract 1 g/L
TBI	$NaNO_3$ 3 g/L, KH_2PO_4 1 g/L, $MgSO_4 \cdot 7H_2O$ 0.5 g/L, KCl 0.5 g/L, $FeSO_4$ 0.01 g/L, sucrose 30 g/L, peptone 10 g/L
SYM	starch 5 g/L, yeast extract 3 g/L, sucrose 1.5 g/L, agar 20 g/L
MM	sucrose 15 g/L, KH_2PO_4 0.5 g/L, KCL 0.25 g/L, $NaNO_3$ 1 g/L, $MgSO_4 \cdot 7H_2O$ 0.5 g/L, $FeSO_4$ 0.005 g/L, agar 20 g/L
PDA	potato 200 g/L, glucose 20 g/L, agar 20 g/L
Wheat grain	distilled water to regulate wheat grain moisture of 40%
