# Supplementary material for: FgUbiH Is Essential for Vegetative Development, Energy Metabolism, and Antioxidant Activity in Fusarium graminearum
Source: Microorganisms. 2024 Oct 20;12(10):2093. doi: 10.3390/microorganisms12102093 (PMC11509934; doi:10.3390/microorganisms12102093)
Supplement: Supplementary file 1 [file microorganisms-12-02093-s001.zip › Table S3.pdf]

Table S3. Primer sequence used in this study.

Primer name	Sequence（5′-3′）	Amplification length (bp)	Annealing temperature(°C)
KFg11100-1F	CTTTATGCGACCCTTCT	786	46
KFg11100-2R	ACAGGCTAATCCAACATAA		
KFg11100-3F	AGTTTGGACCCTGAAGAT	811	47
KFg11100-4R	TTGTGGTGGTGGGAAGT		
KFg11100-5F	ATGGGGTTCAAGGTTATAGTT	1511	53
KFg11100-6R	CCTAAACACCAAACTTGACAGG		
KFg11100-7F	TTTGTTTGCCGCTCTT	1269	41
H855 R	GCTGATCTGACCAGTTGC		
H856 F	GTCGATGCGACGCAATCGT	1063	47
KFg11100-8R	ATACTGGAAATGGGCTGT		
RFg11100F	CTTGATGTCGTCTCGGCTGTC	234	61
RFg11100R	CTTCTTATCCTGGGTTATTTTGCTG		
QTublinF	GTCAGTGCGGTAACCAAATCGGT	95	60
QTublinR	CTCAGAGGTGCCGTTGTAAACACC		
HYG/F	GGCTTGGCTGGAGCTAGTGGAGGTCAA	765	45
HY/R	GTATTGACCGATTCCTTGCGGTCCGAA		
YG/F	GATGTAGGAGGGCGTGGATATGTCCT	921	53
HYG/R	AACCCGCGGTCGGCATCTACTCTATTC		
11100CF	TTAGTGGTGCGATTGACGAGA	4411	57
11100CR	AACACCAAACTTGACAGGTCG		
R01395F	GGGCTGGTCCTGTTGGTTCT	117	60
R01395R	TGAAGTTGAGCGGGATAGTGC		
R01972F	GAAACAACTTCTGGCACGCTA	278	58
R01972R	CTTGACAGGACAGAAAGCACAGT		
R02392F	CTGCCGCAAAGTCGGGTAA	264	58
R02392R	CTTCTGTCTTGATGACGTTGATGT		
R09040F	TCCTACGCTGCCTCATACGC	190	61

Primer name	Sequence（5′-3′）	Amplification length (bp)	Annealing temperature(°C)
R09040R	CGGCTCCTTCGATCAAACC	190	61
R02657F	AGCGTAGCCAACATCCGTG	244	59
R02657R	CCTTCTGGTTATCCTTATTCTTTCG		
R01256F	TCACCCCACAAGGATGATTTC	236	58
R01256R	GGACCAAAAGTGAGCAACAGAT		
R10097F	GTCGCCCAGACCATCAACTC	156	59
R10097R	GAGGGTATCTCGCCAGAACAA		
R02322F	GTCGGTGGCGGTCTTATCA	155	58
R02322R	CCCGTAGGCACATCACTTTG		
R02324F	ACTCAATGCTGGCAGTAAGGG	296	59
R02324R	GCTGTCAAGGGCACAAAGAAC		
R05831F	GCGACGGCAGGAATCACAA	103	60
R05831R	CCGCAAACTCAGAAACGAAA		
R02296F	CATCGCCAAGTTTAGCACCG	215	60
R02296R	CACAATCACGACCGAATCCAC		
RTri1F	GCCTTTCTCGTCGTAGTATCCG	175	61
RTri1R	CAGGGGTTGTTGGGTGCTT		
RTri3F	AGCGGCAGCCAAGGATAAC	175	62
RTri3R	GCGAAAGAGGTCCCCGATAAA		
RTri4F	GCGAAAGAGGTCCCCGATAAA	81	58
RTri4R	ATTCTGGATGGGTCAAGAGGTAT		
RTri5F	TCGTTTTGGATGACAGCAGC	168	60
RTri5R	TCGTTTTGGATGACAGCAGC		
RTri6F	CTTATCGCCCTTCCCACCT	299	58
RTri6R	TCCACCCTGCTAAAGACCCT		
RTri8F	CCACCGAATGGGCATCAGA	260	62
RTri8R	ACCCTCAGCAGCGAGAAAGC		
RTri11F	TTTTGCTTCCTCGGGGTCT	91	60
RTri11R	TCTGTTTCCGTGTCAAGTCGC		
RTri12F	GGGCTGGTATTATGGTTCTGG	138	58
RTri12R	TGGCTGCGTTCTTGTTATCTC		
RTri101F	CCAAATCAGTCTCCTCTACCCC	143	60
RTri101R	TTCCCTCGCTAATGCCCTC		
